# Supplementary material for: Phenotypic and Transcriptomics Analyses Reveal Underlying Mechanisms in a Mouse Model of Corneal Bee Sting
Source: Toxins (Basel). 2022 Jul 8;14(7):468. doi: 10.3390/toxins14070468 (PMC9323056; doi:10.3390/toxins14070468)
Supplement: Supplementary file 1 [file toxins-14-00468-s001.zip › toxins-1771931-supplementary.pdf]

Supplementary Materials: Phenotypic and Transcriptomics Analyses Reveal Underlying Mechanisms in a Mouse Model of Corneal Bee Sting

Yanzi Wang, Honghua Kang, Mengyi Jin, Guoliang Wang, Weifang Ma, Zhen Liu, Yuhua Xue and Cheng Li

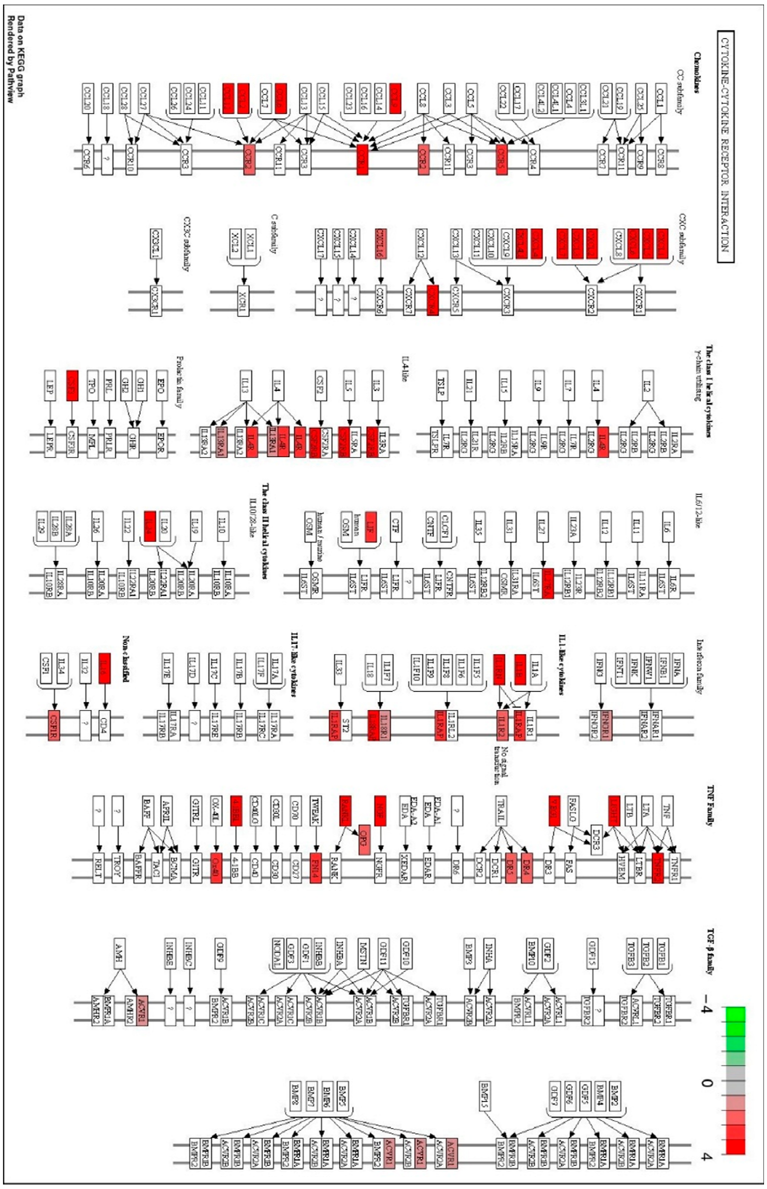

Figure S1. Differences in the most significant signaling pathways. Gene expression in cytokine–cytokine receptor interaction pathways was mapped using PATHVIEW. Red: upregulated genes; green: downregulated genes.
